# Assessment of the Cost of the Mediterranean Diet in a Low-Income Region: Adherence and Relationship with Available Incomes

**DOI:** 10.1186/s12889-021-12433-w

**Published:** 2022-01-10

**Authors:** Alessia Rubini, Cristina Vilaplana-Prieto, Marta Flor-Alemany, Lorena Yeguas-Rosa, Miriam Hernández-González, Francisco Javier Félix-García, Francisco Javier Félix-Redondo, Daniel Fernández-Bergés

**Affiliations:** 1 Research Unit of Don Benito-Villanueva de la Serena Health Area, Calle Sierra Nevada, 10, 06700 Villanueva de la Serena, Spain; 2 University institute for Biosanitary Research of Extremadura (INUBE), Badajoz, Spain; 3 Faculty of Economics and Business, University of Murcia, Murcia, Spain; 4 Department of Physiology, University of Granada, Granada, Spain; 5 Finance and Accounting, University of Extremadura, Badajoz, Spain; 6 Medical Manager of the General Directorate of Health Assistance, Extremadura Health Service, Mérida, Spain

**Keywords:** Mediterranean diet, Food expenditure, Adherence, Monetary cost

## Abstract

**Background:**

The Mediterranean Diet (MD) is recognized as heart-healthy, but the economic cost associated with this type of diet has scarcely been studied.

The objective of the present study is to explore the cost and adherence of a low-income region population to the MD and its relationship with income.

**Methods:**

A population-based study was carried out on 2,833 subjects between 25 and 79 years of age, 54% women, selected at random from the municipalities of Vegas Altas, La Siberia and La Serena in the province of Badajoz, Extremadura (Spain).

Average monthly cost of each product included in the MD was computed and related to adherence to the MD using the Panagiotakos Index and average disposable income.

**Results:**

The monthly median cost was 203.6€ (IQR: 154.04-265.37). Food-related expenditure was higher for men (p<0.001), age cohort between 45 and 54 years (p<0.013) and those living in urban areas (p<0.001). A positive correlation between food-related expenditure and the MD adherence was found. Monthly median cost represents 15% of average disposable income, ranging between 11% for the group with low MD adherence and 17% for the group with high MD adherence.

**Conclusions:**

The monthly cost of the MD was positively correlated with the degree of adherence to this dietary pattern. Given that the estimated monthly cost is similar to that of other Spanish regions with a higher income level, the economic effort required to be able to afford the Mediterranean diet is higher. This may represent a barrier to access, which should be analyzed in detail by public decision-makers.

**Supplementary Information:**

The online version contains supplementary material available at 10.1186/s12889-021-12433-w.

## Introduction and Objectives

Cardiovascular disease (CVD) is the primary cause of death in developed countries. Leading a healthy lifestyle (a proper diet, suitable levels of physical activity, and the reduction of toxic habits) is essential to prevent it [1, 2]. The Mediterranean Diet (MD) is widely recognised as being cardiovascular-healthy [3–5] although few studies have been carried out on the associated cost of this type of food and is rarely used as an argument in the debate on public monitoring by health policy makers.

The MD focuses on the consumption of olive oil as the main source of fat; a high consumption of fruit, vegetables, nuts and dried fruit, and whole grains; a moderate consumption of wine, meat, fish, eggs, and dairy products; and a low consumption of red meat, processed foods, and sugary drinks [1]. Several studies have found that populations with a high adherence to MD patterns show a lower rate of CVD [3–6]. In 2010, UNESCO declared MD an Intangible Cultural Heritage of Humanity and the World Health Organisation classified it as a diet that protects against CVD [7].

The cost of food plays an important role in determining decisions related to the purchase and food consumption [8]. The first evidence on the monetary cost of food products was conducted in the late 1990s [9]. Since then, several studies have reported on the economics of dietary patterns [10–15]. It has been pointed out that healthy diets cost more than unhealthy diets [10]. A recent review showed that a diet rich in fruits, vegetables and nuts was, on average, $1.5 more expensive per day than a diet of processed foods, meat and refined grains [11].

Following a cheaper diet can lead to health problems for consumers due to reduced nutritional quality. Therefore, dietary cost can be a barrier to the adoption of a healthy diet, particularly among people with lower socio-economic status [16, 17].

This socioeconomic gradient in the quality of their diet is therefore likely to contribute to the health disparity among different socioeconomic groups [12], ultimately leading to an increase in overall societal costs [13, 14]. Estimates for Spain have also confirmed the higher cost associated with higher adherence to MD, but remarkable differences can be found between studies, with the cost of high adherence being between 18% and 28% higher than that of low adherence [15, 18]. However, none of these studies have related the cost of MD to disposable income, which is very relevant if we want to assess the affordability of MD for families.

Our objective was to study the cost and level of adherence to the MD of the population of a low-income region, as well as its relationship with the level of disposable income.

## Material and Methods

### Study sample

A cohort study consisting of a population sample of 2,833 subjects selected randomly as representative of between 25 and 79 years of age of the municipalities of Vegas Altas, La Siberia, and La Serena in the province of Badajoz (Extremadura), which amounts to 75,455 inhabitants.

The region of Extremadura was chosen for several reasons. Firstly, because it has one of the highest rates of morbidity and mortality from cardiovascular disease compared to other Spanish regions [19]. Secondly, because it is the Spanish region with the lowest income level (25.82% below the average income) [20].

The methodology used has previously been validated and published [21]. Briefly, the cohort is a population-based study representative of a health area in Extremadura. This was obtained by the simple random method from a database, of the public health service with universal coverage (99.4%). The sample size was calculated to be necessary to estimate the prevalence of different characteristics of the population, with a maximum degree of uncertainty and an accuracy of 2%, resulting 2400 subjects. The inclusion criteria were people between 25 and 79 years old, who lived in one of the 16 populations of more than 2000 inhabitants of the health area, in 2007. Pregnant women, institutionalized subjects, those with serious or terminal illness, and those who were not resident in the afore mentioned area were excluded. The socio demographic profile is described in Table 1.

**Table 1:** Socio demographic profile of the Hermex study

	*Global*	*Male*	*Female*	*p-value*
*N (%)*	2,833 (100%)	1,317 (46.5%)	1,516(53.5%)	
*Age (years), Mean (SD)*	51.2 (14.7)	51.3 (14.6)	51.1 (14.9)	0,734
*Age, distribution by decades*				
25-34	407 (14.4%)	178 (13.5%)	229 (15.1%)	
35-44	671 (23.7%)	309 (23.5%)	362 (23.9%)	
45-54	616 (21.7%)	298 (22.6%)	318 (21.0%)	0.404
55-64	502 (17.7%)	240 (18.2%)	262 (17.3%)	
65-74	418 (14.8%)	201 (15.3%)	217 (14.3%)	
75-79	219 (7.7%)	91 (6.9%)	128 (8.4%)	
*Rural origin*	1,445 (51.0%)	688 (52.2%)	757 (49.9%)	0.221
*Level of education*				
Illiterate	358 (12.7%)	138 (10.5%)	220 (14.6%)	
Primary studies	1,526 (54.2%)	743 (56.8%)	783 (51.9%)	< 0.001
Secondary studies	586 (20.8%)	288 (22.0%)	298 (19.7%)	
Higher degree	348 (12.3%)	140 (10.7%)	208 (13.8%)	

*Source: Félix-Redondo FJ, Fernández-Bergés D, Pérez JF et al. Prevalencia, detección, tratamiento y grado de control de los factores de riesgo cardiovascular en la población de Extremadura. Estudio HERMEX. Aten Primaria. 2011; 43(8) 426-434*

### Nutritional register

The dietary assessment of the participants was carried out using of a previously validated semi-quantitative questionnaire on the frequency of food consumption with 157 food variables and 7 variables related to the consumption of alcoholic drinks [22] (see Table A1 of the Appendix). For all of them, intake was assessed on a scale ranging between 0 and 9, in which 0 corresponds to “never or less than once a month” and 9 to “6 or more times a day”.

### Cost assessment

In order to compute the daily (or monthly) cost of the diet, we have followed three steps. Firstly, records on consumption frequencies were converted to grams or millilitres (see note to Table A1 of the Appendix). Secondly, taking the year 2019 as a baseline, the average price was obtained for each category, using price comparators of supermarkets located in the same geographical area as the study population, i.e. Carrefour, Mercadona, and Día (see Table A2 of the Appendix for details of the number of references used in each category and the average, minimum, and maximum prices). Thirdly, the total daily (or monthly) cost for each participant was obtained as the product of the daily (or monthly) quantity consumed by the price of the respective category.

The costs of the products are expressed indicating the mean and standard deviation (SD) when they follow a Gaussian distribution, and with the median and interquartile range (IQR) when the distribution is not normal.

### Statistical analysis

Once the total cost had been calculated, population was segmented by sex, age, educational level and place of residence. A lineal regression analysis was performed using monthly cost as dependent variable and segmentation variables as regressors (p-values are shown by means of the 3000 bootstrap samples).

Finally, the Panagiotakos Adherence Index (PAI) was used to obtain the correlation between monthly cost and adherence to MD. PAI is a score based on 11 components (non-refined cereals, vegetables, fruit, potatoes, legumes, olive oil, fish, red meat, poultry, full fat dairy products and alcohol). Each component is assigned a score from 0 to 5, depending on intake. Products that are not typical of MD (e.g. red meat) are evaluated on an upside down ladder. The total score ranges from 0 to 55 points, where a higher score leads to a higher level of adherence [23].

Results of PAI were divided distinguishing 3 adherence groups based on the resulting score: low adherence to MD (PAI< 29.99), medium (PAI between 30 and 33.99) and high (PAI >34). A recent systematic review ranks this index as one of those that provides the most outstanding amount of information and for that reason, it has been selected to assess adherence to MD [24].

The different cost levels associated to the different degrees of adherence to MD were compared using the Pearson´s correlation index as a measure of lineal association and the Kruskal-Wallis test as a non-parametric tool to study the significance of the differences between the levels of adherence. A value of p<0.05 was considered significant.

In order to compare monthly cost with income, data from the Statistical System of Extremadura [25] were used. The average disposable income (monthly and annual) was obtained as a weighted average among the different municipalities of the of Vegas Alta, La Serena and La Siberia.

Microsoft Excel and statistical package IBM SPSS 21 were used as computer support.

## Results

Table 2 shows that the median of the monthly food cost (MFC) was 203.63€ (IQR: 154.04-265.37). With a significance level (p<0.001), MFC of men was 13.43% higher for men (216.91€; IQR: 167.64-280.13) than for women (191.22€ (IQR: 145.07-248).

**Table 2:** Descriptive statistics of costs categorized by sex, age, level of education and place of residence.

Sex*		Total	Male	Female			
N		2.833	1.317	1.516			
	%	100%	46,49%	53,51%			
Median		203,63	216,91	191,22			
Percentiles	25	154,04	167,64	145,07			
	75	265,37	280,13	248,00			
Age **		25-34 years	35-44 years	45-54 years	55-64 years	65-74 years	75-79 years
N		407	671	616	502	418	219
	%	14,37%	23,69%	21,74%	17,72%	14,75%	7,73%
Median		203,27	206,63	212,11	210,76	193,95	179,00
Percentiles	25	152,48	158,10	155,47	163,44	149,46	130,21
	75	266,73	270,79	274,63	267,36	246,18	224,99
Place of Residence ***		Rural	Urban				
N		1.445	1.388				
	%	51,36%	48,64%				
Median		188,26	223,52				
Percentiles	25	147,83	166,06				
	75	232,93	296,91				
Level of education ****		Illiterate	Primary studies	Secondary studies	Higher degree, university o similar		
N		358	1.541	586	348		
	%	12,64%	54,39%	20,68%	12,29%		
Median		177,70	208,18	206,57	205,70		
Percentiles	25	131,84	156,83	157,75	161,12		
	75	223,73	269,78	271,28	269,06		

Own elaboration with data from HERMEX study. * p <0,001; ** p <0,013; *** p < 0,001; **** p < 0,701

The MFC median by age cohorts (p<0.013) has an inverted U-shape with a maximum attained for the cohort aged 45-54 years (212.11€; IQR 155.47-274.63). The minimum cost corresponds to the cohort of 75-79 years (179€; IQR 130.21-224.99), i.e. 15.61% less than the cohort aged 45-54 years old.

As for the place of residence, the cost in rural areas was 188.26€ (IQR: 147.83 -232.93) as compared to 223.52€ (IQR; 166.06 -296.91) in urban areas, i.e. 14,64% less among residents in rural areas (p<0.001).

**Fig. 1:**
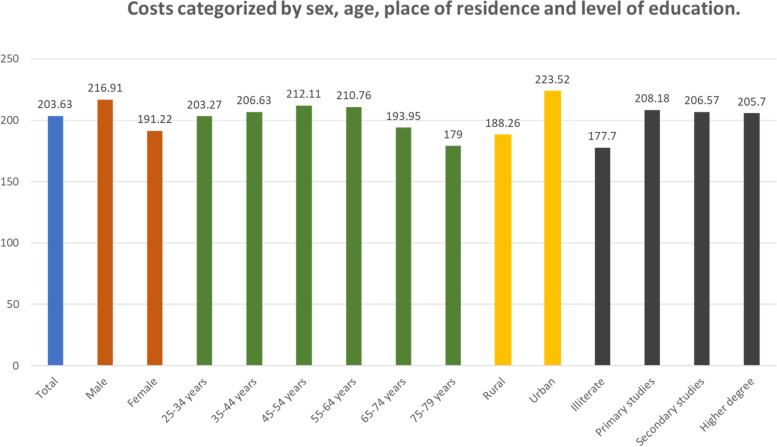
Costs categorized by sex, age, place of residence and level of education

No significant differences (*p*<0.701) were found between different levels of education and the MFC (see Fig. 1).

As for adherence to MD, the higher adherence, the higher MFC. According to PAI, MFC associated with high adherence was 229.38€, that is, 34.50% and 49.37% higher as compared to medium and low adherence, respectively. The positive values of the Pearson coefficient and the significant differences between the different adherence groups according to the Kruskal-Wallis test support these results. (Table 3).

**Table 3:** Monthly cost according to the indicator of adherence to the Mediterranean Diet of Panagiotakos

		Low adherence	Medium adherence	High adherence
N		335	828	1670
	%	11,82	29,23	59,95
Median		153,56	170,54	229,38
Percentiles	25	117,25	138,46	184,35
	75	204,19	213,30	295,64
Pearson's correlation		0,39		
Kruskal-Wallis’ test		0,001		

Own elaboration with data from HERMEX study

Table 4 shows the MFC and the PAI as percentages of various income indicators. The median of the MFC represents 22.60% of the official minimum wage, 37.81% of the IPREM (indicator used to calculate the minimum guaranteed income by social benefits), 44.02% of the minimum living income support, and 51.88% of the non-contributory pension. Even for low adherence to MD, monthly cost is close to 30% of the IPREM and above this threshold for minimum living income support and non-contributory pensions.

**Table 4:** Monthly cost and adherence rates for various income indicators

	Median cost (€/month)	MW * 2019	IPREM** 2019	MVI *** 2020	nCP **** 2019
Income index (€/month)		900	537,84	462	392
In % respect to the average income	203,36	22,60%	37,81%	44,02%	51,88%
PAI*****					
Low adherence	153,56	17,06%	28,55%	33,24%	39,17%
Medium adherence	170,54	18,95%	31,71%	36,91%	43,51%
High adherence	229,38	25,49%	42,65%	49,65%	58,52%
	Median cost (€/month)	Spain	Extremadura	Badajoz	Municipalities of Vegas Altas, La Siberia and La Serena
Average disposable income (€/year)		22.634	17.361	17.439	16.288
Average disposable income (€/month)		1886,17	1446,75	1453,25	1357,30
In % respect to the average income	203,36	10,78%	14,06%	13,99%	14,98%
PAI*****					
Low adherence	153,56	8,14%	10,61%	10,57%	11,31%
Medium adherence	170,54	9,04%	11,79%	11,74%	12,56%
High adherence	229,38	12,16%	15,85%	15,78%	16,90%

* MW: Minimum Wage

** IPREM: Public Indicator of Multiple Effects Income

*** MVI: Minimum Living Income (single-person household)

**** nCP: Non-Contributory Pension (one-person household)

***** PAI: Panagiotakos Adherence Index

Own elaboration based on data from the Statistical System of Extremadura - Citizen Portal (gobex.es)

Table 4 exhibits the MFC and PAI with respect to the mean disposable income for Spain, Extremadura, Badajoz and in the municipalities of Vegas Altas, La Siberia and La Serena. In the aforementioned municipalities, the MFC amounts to 14.98% of the average disposable income, ranging between 11.31% for the group with low adherence and 16.90% for the group with high adherence. These values are similar for Badajoz and Extremadura. At the country level, MFC represents 10.78% of the national mean disposable income, ranging from 8.14% for low adherence group and 12.16% for high adherence group.

## Discussion

The main result is that the average monthly food cost (MFC) shows a positive correlation with level of adherence to MD and amounts to 203.63€. In second place, we highlight that MFC is higher for men, the cohort aged between 45 and 54 years and those living in urban areas.

This study is the first to relate the cost, level of adherence, and level of income to the MD in the population of Extremadura, which is that of the lowest per capita income in Spain [20].

### Costs, economic resources, and adherence to the MD

Despite the significant income differences among Spanish regions, such as Navarra, Cataluña, and Extremadura [20], our estimated MFC is coherent with previous research by Fresán et al. [26] and Schröder et al [18]. The former study relied on university student data aged between 27 and 45 years old living in Catalonia (whose average income is 14.40% above the Spanish mean [20]) found that the estimated MFC was 226€. In the study by Schröder et al [18], which focused on the aged cohort between 25 and 74 years of age living in Navarra (where average income is 18.81% above the Spanish mean [20]), the MFC was 220€. Considering that the average levels of income of the regions are very different, it is clear that the economic effort will differ in accordance with the income of each region.

The relationship between economic resources and the level of adherence to a healthy diet is a factor which is not usually considered in daily welfare clinical practice. Various studies [10, 15, 16] have described a higher level of adherence to the healthy pattern of the MD in the population with higher income. Schröder et al. [18] observed that the MFC associated with high adherence was 237.60€, while that associated with low adherence was 202.20€.

On the other side, López et al. [27] stressed that a good adherence to the MD involved a higher cost than good adherence to other dietary patterns. In their study they described the eating behaviour of 11,195 participants and concluded that the expenditure of individuals with good adherence to the MD was higher (0.64€ more for each 1000 kcal) as compared to those with the same level of adherence following the “Western food pattern”.

According to a study of 2014 by the Harvard School of Public Health [28], which analysed consumer choices in recent decades, differences in the choice of food between individuals belonging to extreme income deciles are becoming more evident every year. The economic crisis contributed towards the increasingly unhealthy eating habits of the population with lower economic resources. This study also revealed significant differences according to educational attainment. However, this variable was not relevant in our research.

The Moli-Sani study [29], analyzed the relationship between adherence to healthy diet and economic characteristics of the adult population living in Italian region of Molise. The higher income groups showed a positive difference of two points in adherence to the MD, which was also associated with a 15% reduction of the risk of CVD.

So far, the sex perspective has not been considered in most studies that have addressed the cost of diet, especially in the case of adherence to the MD. In our study, the MFC of women's diets is 11.84% lower than men's. Underlying reasons may involve lower income level of women [30, 31], differences in dietary patterns, as well as a better use of resources due to the socio-cultural pattern that has determined deeper involvement in household chores.

Neither have we found any information on lower expenses in rural areas; in our opinion this may be due to the lower cost of foods in this milieu.

Finally, the MFC is 15.61% lower in the 75-59 age cohort than in the 45-54 age cohort. There is evidence that older cohorts stick more closely to Mediterranean dietary pattern in their meals [32].

A closer look should be taken at all this in suitably designed studies.

### Choices in the management of public health

From the perspective of public health policies, our results strengthen the importance of strategies towards the improvement of diet quality, especially, focusing on the lowest socioeconomic groups [33]. These actions should be implemented in two complementary fields. Firstly, consumers should be educated on the nutritional value and cost of food. In this sense the studies of Drewnowski et al. [34] and Goulet et al. [35] confirm the fact that Mediterranean-style foods can be obtained at all price ranges whether in grams or in calories. The only condition for keeping the Mediterranean diet pattern at a reasonable price is to include more cereals, vegetables, and seasonal fruit. Therefore, emphasis should be placed on communication strategies that disseminate how healthy foods can be selectively purchased within products of different economic value at a lower total cost.

Secondly, there is a controverted debate about the reduction of indirect taxes (VAT) on healthy (e.g. MD) [36, 37], and/or the increase of taxes on unhealthy food (e.g. sugary drinks) [38–40] is being discussed. In this sense, Andreyeva et al [41], highlight the difficulty to carry out a rigorous analysis because changes in the economic scenario (i.e., increase of VAT) could alter price and cross demand elasticities.

Future research should focus on the prediction of the impact of these fiscal policies and in this sense, interdisciplinary work is essential in order to reach a consensus.

## Limitations

The main limitations faced by this work are twofold. On the one hand, the fact of using consumption data corresponding to the period 2007-2009 and price data for 2019. Secondly, the possibility of extrapolating the results obtained for Extremadura to the national level.

With regard to the first limitation, table A3 in the Appendix shows the average annual cumulative rate of the Consumer Price Index (CPI) for food, non-alcoholic beverages and alcoholic beverages, as well as the average disposable income in the period 2008-2019. It can be seen that the cumulative average annual growth rate in Badajoz is slightly higher than the growth rate of the aforementioned CPI components. Therefore, there has been no loss of purchasing power in these components over the period considered, and the dietary data collected in 2007-2009 could be considered representative of the diet in 2019.

Regarding the second limitation, the extrapolation of the average cost of the MD in the municipalities of Vegas Alta, La Serena and La Siberia to the country as a whole presents some restrictions. Firstly, the average disposable income in Spain was 37.67% (29.79%) higher than in Badajoz in 2008 (2019).

Secondly, the percentage of household expenditure devoted to food is higher in Badajoz (39.20 percentage points in 2008 and 42.27 percentage points in 2019) (see table A4 in the Appendix). However, for the whole country and for Badajoz, there has been a decrease in the share of food in the shopping basket (-13.96 points for Spain and -17.03 points for Badajoz). On the other hand, the average annual accumulated growth rate of average disposable income has been higher in Badajoz (1.376% compared to 0.834%) and also higher than the growth of the three groups of goods considered (food, non-alcoholic beverages and alcoholic beverages) (see Table A4 in the Appendix). These data suggest a process of convergence of average disposable income in Badajoz to the Spanish average income and also a greater similarity with average consumption patterns at the national level.

## Conclusions

The MFC in a low-income region was positively correlated with the level of adherence to the MD and is similar to other Spanish regions with higher per capita income, which relates higher expenditure to achieve equality of adherence to the MD.

With regard to the measures traditionally proposed (taxes or subsidies), it should be considered an implementation of these, at regional level (in the poorest regions, where access to MD is less affordable in relative terms) for example in the form of income supplements for the lowest levels of income. In any case, further research on the cost-effectiveness of these measures and coordination in fiscal and social policies would be necessary.

The sex deserves a more in-depth analysis.

Knowledge and handling of this information by healthcare personnel should be useful if they consider in their dietary indications the economic factors having a positive or negative effect on their compliance.

## Supplementary Information

**Additional file 1 **: **Table A1**. Amounts consumed depending on the frequency of consumption. **Table A2**. Descriptive statistics for the prices of all product categories. **Table A3**. Comparison of evolution of food and beverages prices and average disposable income in the period 2008-2019. **Table A4**. Comparison of the weight that food and beverages represent in the shopping basket.

## Data Availability

Data available: Rubini, Alessia (2021): HERMEX BMC.sav. figshare. Dataset. 10.6084/m9.figshare.17104760.v1.

## List of abbreviations

CVD: Cardiovascular disease
MD: Mediterranean Diet
PAI: Panagiotakos Adherence Index
MFC: Monthly Food Cost
