# Correction to: Assessment of the cost of the Mediterranean diet in a low-income region: adherence and relationship with available incomes

**DOI:** 10.1186/s12889-022-12583-5

**Published:** 2022-01-21

**Authors:** Alessia Rubini, Cristina Vilaplana-Prieto, Marta Flor-Alemany, Lorena Yeguas-Rosa, Miriam Hernández-González, Francisco Javier Félix-García, Francisco Javier Félix-Redondo, Daniel Fernández-Bergés

**Affiliations:** 1 Research Unit of Don Benito-Villanueva de la Serena Health Area, Calle Sierra Nevada, 10, 06700 Villanueva de la Serena, Spain; 2 University institute for Biosani- tary Research of Extremadura (INUBE), Badajoz, Spain; 3 Faculty of Economics and Business, University of Murcia, Murcia, Spain; 4 Department of Physiology, University of Granada, Granada, Spain; 5 Finance and Accounting, University of Extremadura, Badajoz, Spain; 6 Medical Manager of the General Directorate of Health Assistance, Extremadura Health Service, Mérida, Spain

**Correction to: BMC Public Health 22, 58 (2022)**

**https://doi.org/10.1186/s12889-021-12433-w**

In the original publication [1], there was an incorrect funding section. The incorrect and correct funding information is published in this correction article, the updated information is shown in bold. The original article has been updated.

**Incorrect funding**

The present work has been financed by: Carlos III Health Institute [PI071218 National Plan for Scientific Research 2004–07 (Funded the medical staff for 3 years and material for the extraction of samples and indirect costs of the project) - EMER 07/046 Grants for emerging research groups (Funding of personnel: nurse, doctor and technician) - INT 07/289 Grants for intensification of personal care research (Funded a researcher for 6 months)], Fundesalud [Integral Plan Grant for Cardiovascular Diseases in Extremadura (Funded a lab technician during patient enrolment)] and Junta de Extremadura and European Union (ERDF) [GR18076 Aid to Research Groups 2018 (Funded a research support technician)].

**Correct funding**

The present work has been financed by: Carlos III Health Institute [PI071218 National Plan for Scientific Research 2004–07 (Funded the medical staff for 3 years and material for the extraction of samples and indirect costs of the project) - EMER 07/046 Grants for emerging research groups (Funding of personnel: nurse, doctor and technician) - INT 07/289 Grants for intensification of personal care research (Funded a researcher for 6 months)], Fundesalud [Integral Plan Grant for Cardiovascular Diseases in Extremadura (Funded a lab technician during patient enrolment)] and Junta de Extremadura and European Union (ERDF) [GR18076 Aid to Research Groups 2018 (Funded a research support technician) and GR21015 Aid to Research Groups 2021]. We also thank Spain’s Ministry of Science, Innovation and Universities (MICINN) and the ERDF for financial support: PID2020-114231RB-I00 and RTI2018–095256-BI00.
