# Supplementary material for: Assessment of the Cost of the Mediterranean Diet in a Low-Income Region: Adherence and Relationship with Available Incomes
Source: BMC Public Health. 2022 Jan 10;22:58. doi: 10.1186/s12889-021-12433-w (PMC8751306; doi:10.1186/s12889-021-12433-w)
Supplement: Supplementary file 1 — Additional file 1 : Table A1. Amounts consumed depending on the frequency of consumption. Table A2. Descriptive statistics for the prices of all product categories. Table A3. Comparison of evolution of food and beverages prices and average disposable income in the period 2008-2019. Table A4. Comparison of the weight that food and beverages represent in the shopping basket. [file 12889_2021_12433_MOESM1_ESM.docx]

**Assessment of the Cost of the Mediterranean Diet in a Low-Income Region: Adherence and Relationship with Available Incomes**

**Complementary Tables. Appendix**

**Table A1. Amounts consumed depending on the frequency of consumption.**

|  | Times a month | | Times a week | | | | Times a day | | | | Portion size (g/ml) |
| --- | --- | --- | --- | --- | --- | --- | --- | --- | --- | --- | --- |
|  | Less than 1 | 1-3 | 1 | 1-2 | 3-4 | 5-6 | 1 | 2-3 | 4-5 | 6+ |  |
| Sunflower oil (one tablespoon) | 0 | 0.90 | 19.31 | 2.89 | 6.75 | 10.61 | 13.5 | 33.75 | 60.75 | 81 | 13.5 |
| Corn oil (one dessertspoon) | 0 | 0.90 | 19.31 | 2.89 | 6.75 | 10.61 | 13.5 | 33.75 | 60.75 | 81 | 13.5 |
| Extra virgin olive oil (one tablespoon) | 0 | 0.90 | 19.31 | 2.89 | 6.75 | 10.61 | 13.5 | 33.75 | 60.75 | 81 | 13.5 |
| Olive oil (one tablespoon) | 0 | 0.90 | 19.31 | 2.89 | 6.75 | 10.61 | 13.5 | 33.75 | 60.75 | 81 | 13.5 |
| Olive oil (pomace) (one tablespoon) | 0 | 0.90 | 19.31 | 2.89 | 6.75 | 10.61 | 13.5 | 33.75 | 60.75 | 81 | 13.5 |
| Swiss chard (a normal dish) | 0 | 11.73 | 25.03 | 37.45 | 87.50 | 137.55 | 175 | 437.5 | 787.5 | 1050 | 175 |
| Avocado (one piece) | 0 | 13.40 | 28.60 | 42.80 | 100.00 | 157.20 | 200 | 500 | 900 | 1200 | 200 |
| Clams (a normal can) | 0 | 2.68 | 5.72 | 8.56 | 20.00 | 31.44 | 40 | 100 | 180 | 240 | 40 |
| Shelled almonds (a handful. 30g) | 0 | 2.01 | 4.29 | 6.42 | 15.00 | 23.58 | 30 | 75 | 135 | 180 | 30 |
| Fruits in syrup (a dessert plate) | 0 | 8.04 | 17.16 | 25.68 | 60.00 | 94.32 | 120 | 300 | 540 | 720 | 120 |
| Anchovies in oil (a normal can) | 0 | 2.68 | 5.72 | 8.56 | 20.00 | 31.44 | 40 | 100 | 180 | 240 | 40 |
| White rice (a normal dish) | 0 | 13.40 | 28.60 | 42.80 | 100.00 | 157.20 | 200 | 500 | 900 | 1200 | 200 |
| Brown rice (a normal dish) | 0 | 13.40 | 28.60 | 42.80 | 100.00 | 157.20 | 200 | 500 | 900 | 1200 | 200 |
| Tuna. mackerel. bonito in oil (a normal can) | 0 | 6.16 | 13.16 | 19.69 | 46.00 | 72.31 | 92 | 230 | 414 | 552 | 92 |
| Pickle Tuna, mackerel or bonito (a normal can) | 0 | 6.16 | 13.16 | 19.69 | 46.00 | 72.31 | 92 | 230 | 414 | 552 | 92 |
| Fresh tuna (one slice) | 0 | 10.05 | 21.45 | 32.10 | 75.00 | 117.90 | 150 | 375 | 675 | 900 | 150 |
| Shelled hazelnuts (a handful. 30g) | 0 | 2.01 | 4.29 | 6.42 | 15.00 | 23.58 | 30 | 75 | 135 | 180 | 30 |
| Oat flakes (one bowl) | 0 | 6.30 | 13.44 | 20.12 | 47.00 | 73.88 | 94 | 235 | 423 | 564 | 94 |
| Sugar (one teaspoon) | 0 | 0.34 | 0.72 | 1.21 | 2.50 | 3.93 | 5 | 12.5 | 22.5 | 30 | 5 |
| Bacon (two slices) | 0 | 2.01 | 4.29 | 6.42 | 15.00 | 23.58 | 30 | 75 | 135 | 180 | 30 |
| Carbonated drinks- Soft drink: Coke, Fanta and similar (one glass. 200 ml) | 0 | 13.40 | 28.60 | 42.80 | 100.00 | 157.20 | 200 | 500 | 900 | 1200 | 200 |
| Eggplants, zucchini (a normal dish) | 0 | 11.73 | 25.03 | 37.45 | 87.50 | 137.55 | 175 | 437.5 | 787.5 | 1050 | 175 |
| Brie or camembert (a 50g cut) | 0 | 3.35 | 7.15 | 10.70 | 25.00 | 39.30 | 50 | 125 | 225 | 300 | 50 |
| Broccoli (a normal dish) | 0 | 11.73 | 25.03 | 37.45 | 87.50 | 137.55 | 175 | 437.5 | 787.5 | 1050 | 175 |
| Beef (a steak) | 0 | 10.05 | 21.45 | 32.10 | 75.00 | 117.90 | 150 | 375 | 675 | 900 | 150 |
| Blood sausage | 0 | 10.05 | 21.45 | 32.10 | 75.00 | 117.90 | 150 | 375 | 675 | 900 | 150 |
| Mackerel or anchovy (one dish) | 0 | 10.05 | 21.45 | 32.10 | 75.00 | 117.90 | 150 | 375 | 675 | 900 | 150 |
| Shelled peanuts (a handful. 30g) | 0 | 2.01 | 4.29 | 6.42 | 15.00 | 23.58 | 30 | 75 | 135 | 180 | 30 |
| Coffee with milk (a large cup) | 0 | 10.05 | 21.45 | 32.10 | 75.00 | 117.90 | 150 | 375 | 675 | 900 | 150 |
| Black coffee (one cup) | 0 | 3.35 | 7.15 | 10.70 | 25.00 | 39.30 | 50 | 125 | 225 | 300 | 50 |
| Squid (a normal dish) | 0 | 10.05 | 21.45 | 32.10 | 75.00 | 117.90 | 150 | 375 | 675 | 900 | 150 |
| Cannelloni (a normal dish) | 0 | 13.40 | 28.60 | 42.80 | 100.00 | 157.20 | 200 | 500 | 900 | 1200 | 200 |
| Sparkling wine | 0 | 10.05 | 21.45 | 32.10 | 75.00 | 117.90 | 150 | 375 | 675 | 900 | 150 |
| Onions (one small onion. 100g) | 0 | 6.70 | 14.30 | 21.40 | 50.00 | 78.60 | 100 | 250 | 450 | 600 | 100 |
| Fatty pork (ribs. chops) | 0 | 10.05 | 21.45 | 32.10 | 75.00 | 117.90 | 150 | 375 | 675 | 900 | 150 |
| Lean pork (tenderloin. sirloin) | 0 | 10.05 | 21.45 | 32.10 | 75.00 | 117.90 | 150 | 375 | 675 | 900 | 150 |
| Beer with alcohol (one half. 330ml) | 0 | 22.11 | 47.19 | 70.62 | 165.00 | 259.38 | 330 | 825 | 1485 | 1980 | 330 |
| Non-alcoholic beer (one half. 330ml) | 0 | 22.11 | 47.19 | 70.62 | 165.00 | 259.38 | 330 | 825 | 1485 | 1980 | 330 |
| Chocolate (one chocolate bar. 20g) | 0 | 1.34 | 2.86 | 4.28 | 10.00 | 15.72 | 20 | 50 | 90 | 120 | 20 |
| Plums (one piece) | 0 | 4.42 | 9.44 | 14.12 | 33.00 | 51.88 | 66 | 165 | 297 | 396 | 66 |
| Cabbage (a normal dish) | 0 | 11.73 | 25.03 | 37.45 | 87.50 | 137.55 | 175 | 437.5 | 787.5 | 1050 | 175 |
| Brussels sprouts (a normal dish) | 0 | 11.73 | 25.03 | 37.45 | 87.50 | 137.55 | 175 | 437.5 | 787.5 | 1050 | 175 |
| Hot chocolate. cocoa powder etc. (a large cup) | 0 | 13.40 | 28.60 | 42.80 | 100.00 | 157.20 | 200 | 500 | 900 | 1200 | 200 |
| Rabbit (one quarter) | 0 | 10.05 | 21.45 | 32.10 | 75.00 | 117.90 | 150 | 375 | 675 | 900 | 150 |
| Lamb meat (one plate) | 0 | 10.05 | 21.45 | 32.10 | 75.00 | 117.90 | 150 | 375 | 675 | 900 | 150 |
| Corn flakes (one bowl) | 0 | 1.94 | 4.15 | 6.21 | 14.50 | 22.79 | 29 | 72.5 | 130.5 | 174 | 29 |
| Croissant (one) | 0 | 3.35 | 7.15 | 10.70 | 25.00 | 39.30 | 50 | 125 | 225 | 300 | 50 |
| Croquettes (three units) | 0 | 10.05 | 21.45 | 32.10 | 75.00 | 117.90 | 150 | 375 | 675 | 900 | 150 |
| Dates, raisins, dried figs (one serving. 100 g) | 0 | 6.70 | 14.30 | 21.40 | 50.00 | 78.60 | 100 | 250 | 450 | 600 | 100 |
| Donut (one) | 0 | 3.35 | 7.15 | 10.70 | 25.00 | 39.30 | 50 | 125 | 225 | 300 | 50 |
| Endives (a normal dish) | 0 | 11.73 | 25.03 | 37.45 | 87.50 | 137.55 | 175 | 437.5 | 787.5 | 1050 | 175 |
| Escarole (a normal dish) | 0 | 3.35 | 7.15 | 10.70 | 25.00 | 39.30 | 50 | 125 | 225 | 300 | 50 |
| Fresh asparagus (a normal dish) | 0 | 11.73 | 25.03 | 37.45 | 87.50 | 137.55 | 175 | 437.5 | 787.5 | 1050 | 175 |
| Spinach (a normal dish) | 0 | 13.40 | 28.60 | 42.80 | 100.00 | 157.20 | 200 | 500 | 900 | 1200 | 200 |
| Salami, salchichón (three slices) | 0 | 1.68 | 3.58 | 5.35 | 12.50 | 19.65 | 25 | 62.5 | 112.5 | 150 | 25 |
| Cookies (6 units) | 0 | 2.41 | 5.15 | 7.70 | 18.00 | 28.30 | 36 | 90 | 162 | 216 | 36 |
| Prawns or similar (5 or 6 units) | 0 | 3.35 | 7.15 | 10.70 | 25.00 | 39.30 | 50 | 125 | 225 | 300 | 50 |
| Chickpeas (a normal dish) | 0 | 13.40 | 28.60 | 42.80 | 100.00 | 157.20 | 200 | 500 | 900 | 1200 | 200 |
| Peas (a normal dish) | 0 | 11.73 | 25.03 | 37.45 | 87.50 | 137.55 | 175 | 437.5 | 787.5 | 1050 | 175 |
| Broad beans (a normal dish) | 0 | 13.40 | 28.60 | 42.80 | 100.00 | 157.20 | 200 | 500 | 900 | 1200 | 200 |
| Ice cream (one serving) | 0 | 10.05 | 21.45 | 32.10 | 75.00 | 117.90 | 150 | 375 | 675 | 900 | 150 |
| Beef. pork or lamb liver | 0 | 10.05 | 21.45 | 32.10 | 75.00 | 117.90 | 150 | 375 | 675 | 900 | 150 |
| Cooked ham (one slice) | 0 | 2.01 | 4.29 | 6.42 | 15.00 | 23.58 | 30 | 75 | 135 | 180 | 30 |
| Serrano ham (one slice) | 0 | 2.01 | 4.29 | 6.42 | 15.00 | 23.58 | 30 | 75 | 135 | 180 | 30 |
| Green beans (a normal dish) | 0 | 11.73 | 25.03 | 37.45 | 87.50 | 137.55 | 175 | 437.5 | 787.5 | 1050 | 175 |
| Fried tomato or ketchup (one dessertspoon) | 0 | 0.50 | 1.07 | 1.61 | 3.75 | 5.90 | 7.5 | 18.75 | 33.75 | 45 | 7.5 |
| Kiwi (Two pieces) | 0 | 9.38 | 20.02 | 29.96 | 70.00 | 110.04 | 140 | 350 | 630 | 840 | 140 |
| Skim milk (one large cup) | 0 | 13.40 | 28.60 | 42.80 | 100.00 | 157.20 | 200 | 500 | 900 | 1200 | 200 |
| Whole milk (one large cup) | 0 | 13.40 | 28.60 | 42.80 | 100.00 | 157.20 | 200 | 500 | 900 | 1200 | 200 |
| Semi-skimmed milk (one large cup) | 0 | 13.40 | 28.60 | 42.80 | 100.00 | 157.20 | 200 | 500 | 900 | 1200 | 200 |
| Soy milk (one large cup) | 0 | 13.40 | 28.60 | 42.80 | 100.00 | 157.20 | 200 | 500 | 900 | 1200 | 200 |
| Lentils (a normal dish) | 0 | 13.40 | 28.60 | 42.80 | 100.00 | 157.20 | 200 | 500 | 900 | 1200 | 200 |
| Liquors (50ml) | 0 | 3.35 | 7.15 | 10.70 | 25.00 | 39.30 | 50 | 125 | 225 | 300 | 50 |
| Lemon (one piece) | 0 | 3.89 | 8.29 | 12.41 | 29.00 | 45.59 | 58 | 145 | 261 | 348 | 58 |
| Cupcakes (one) | 0 | 3.35 | 7.15 | 10.70 | 25.00 | 39.30 | 50 | 125 | 225 | 300 | 50 |
| Mayonnaise (a dessertspoon) | 0 | 0.50 | 1.07 | 1.61 | 3.75 | 5.90 | 7.5 | 18.75 | 33.75 | 45 | 7.5 |
| Butter (a dessertspoon) | 0 | 0.94 | 2.00 | 3.00 | 7.00 | 11.00 | 14 | 35 | 63 | 84 | 14 |
| Apple (one piece) | 0 | 13.27 | 28.31 | 42.37 | 99.00 | 155.63 | 198 | 495 | 891 | 1188 | 198 |
| Margarine (one dessertspoon) | 0 | 0.94 | 2.00 | 3.00 | 7.00 | 11.00 | 14 | 35 | 63 | 84 | 14 |
| Mussels, clams or similar (a normal dish) | 0 | 3.35 | 7.15 | 10.70 | 25.00 | 39.30 | 50 | 125 | 225 | 300 | 50 |
| Peach (one piece) | 0 | 5.83 | 12.44 | 18.62 | 43.50 | 68.38 | 87 | 217.5 | 391.5 | 522 | 87 |
| Melon (one cut) | 0 | 10.65 | 22.74 | 34.03 | 79.50 | 124.97 | 159 | 397.5 | 715.5 | 954 | 159 |
| Mortadella, chorizo, Vienna sausage (one slice) | 0 | 1.34 | 2.86 | 4.28 | 10.00 | 15.72 | 20 | 50 | 90 | 120 | 20 |
| Muesli (one bowl) | 0 | 6.30 | 13.44 | 20.12 | 47.00 | 73.88 | 94 | 235 | 423 | 564 | 94 |
| Orange (one piece) | 0 | 17.22 | 36.75 | 55.00 | 128.50 | 202.00 | 257 | 642.5 | 1156.5 | 1542 | 257 |
| Whipped cream. heavy cream (1/2 large cup) | 0 | 2.35 | 5.01 | 7.49 | 17.50 | 27.51 | 35 | 87.5 | 157.5 | 210 | 35 |
| Custard. flan. pudding (one large cup) | 0 | 10.05 | 21.45 | 32.10 | 75.00 | 117.90 | 150 | 375 | 675 | 900 | 150 |
| Eggs (one serving. 50g) | 0 | 3.35 | 7.15 | 10.70 | 25.00 | 39.30 | 50 | 125 | 225 | 300 | 50 |
| Sliced white bread (one slice) | 0 | 1.68 | 3.58 | 5.35 | 12.50 | 19.65 | 25 | 62.5 | 112.5 | 150 | 25 |
| Sliced whole wheat bread (one slice) | 0 | 1.68 | 3.58 | 5.35 | 12.50 | 19.65 | 25 | 62.5 | 112.5 | 150 | 25 |
| Bread (one slice) | 0 | 3.35 | 7.15 | 10.70 | 25.00 | 39.30 | 50 | 125 | 225 | 300 | 50 |
| Pasta (spaghetti. etc.) (a normal dish) | 0 | 13.40 | 28.60 | 42.80 | 100.00 | 157.20 | 200 | 500 | 900 | 1200 | 200 |
| Whole wheat pasta (a normal dish) | 0 | 13.40 | 28.60 | 42.80 | 100.00 | 157.20 | 200 | 500 | 900 | 1200 | 200 |
| Cake (one portion. 150g) | 0 | 10.05 | 21.45 | 32.10 | 75.00 | 117.90 | 150 | 375 | 675 | 900 | 150 |
| Potato chips (a normal bag) | 0 | 2.01 | 4.29 | 6.42 | 15.00 | 23.58 | 30 | 75 | 135 | 180 | 30 |
| Potatoes (one medium potato. 150g) | 0 | 13.40 | 28.60 | 42.80 | 100.00 | 157.20 | 200 | 500 | 900 | 1200 | 200 |
| Pate / foie gras (one serving. 25 g) | 0 | 1.68 | 3.58 | 5.35 | 12.50 | 19.65 | 25 | 62.5 | 112.5 | 150 | 25 |
| Chicken or turkey breast without skin | 0 | 10.05 | 21.45 | 32.10 | 75.00 | 117.90 | 150 | 375 | 675 | 900 | 150 |
| Cucumber (one medium cucumber. 200g) | 0 | 13.40 | 28.60 | 42.80 | 100.00 | 157.20 | 200 | 500 | 900 | 1200 | 200 |
| Pear (one piece) | 0 | 15.95 | 34.03 | 50.93 | 119.00 | 187.07 | 238 | 595 | 1071 | 1428 | 238 |
| White fish: Hake, sole, monkfish etc. (one dish) | 0 | 10.05 | 21.45 | 32.10 | 75.00 | 117.90 | 150 | 375 | 675 | 900 | 150 |
| Peppers (one small pepper. 200g) | 0 | 13.40 | 28.60 | 42.80 | 100.00 | 157.20 | 200 | 500 | 900 | 1200 | 200 |
| Pistachios (one serving. 30g) | 0 | 2.01 | 4.29 | 6.42 | 15.00 | 23.58 | 30 | 75 | 135 | 180 | 30 |
| Banana (one piece) | 0 | 12.80 | 27.31 | 40.87 | 95.50 | 150.13 | 191 | 477.5 | 859.5 | 1146 | 191 |
| Blue cheese (50g) | 0 | 3.35 | 7.15 | 10.70 | 25.00 | 39.30 | 50 | 125 | 225 | 300 | 50 |
| Gruyere / Emmental / gouda (50g) | 0 | 3.35 | 7.15 | 10.70 | 25.00 | 39.30 | 50 | 125 | 225 | 300 | 50 |
| Hard cheese (50g) | 0 | 3.35 | 7.15 | 10.70 | 25.00 | 39.30 | 50 | 125 | 225 | 300 | 50 |
| Cottage cheese, or other soft and fresh cheese (50g) | 0 | 3.35 | 7.15 | 10.70 | 25.00 | 39.30 | 50 | 125 | 225 | 300 | 50 |
| Beef. pork. or lamb kidneys (one dish) | 0 | 10.05 | 21.45 | 32.10 | 75.00 | 117.90 | 150 | 375 | 675 | 900 | 150 |
| Salt (a pinch) | 0 | 0.07 | 0.14 | 0.21 | 0.50 | 0.79 | 1 | 2.5 | 4.5 | 6 | 1 |
| Salmon (one slice) | 0 | 10.05 | 21.45 | 32.10 | 75.00 | 117.90 | 150 | 375 | 675 | 900 | 150 |
| Watermelon (one cut) | 0 | 10.65 | 22.74 | 34.03 | 79.50 | 124.97 | 159 | 397.5 | 715.5 | 954 | 159 |
| Sardines (Six units) | 0 | 10.05 | 21.45 | 32.10 | 75.00 | 117.90 | 150 | 375 | 675 | 900 | 150 |
| Sardines in oil (a normal can) | 0 | 8.38 | 17.88 | 26.75 | 62.50 | 98.25 | 125 | 312.5 | 562.5 | 750 | 125 |
| Pickled sardines (a normal can) | 0 | 8.38 | 17.88 | 26.75 | 62.50 | 98.25 | 125 | 312.5 | 562.5 | 750 | 125 |
| Mushrooms (a normal dish) | 0 | 11.73 | 25.03 | 37.45 | 87.50 | 137.55 | 175 | 437.5 | 787.5 | 1050 | 175 |
| Soup. Broth (a normal dish) | 0 | 26.80 | 57.20 | 85.60 | 200.00 | 314.40 | 400 | 1000 | 1800 | 2400 | 400 |
| Fat beef | 0 | 10.05 | 21.45 | 32.10 | 75.00 | 117.90 | 150 | 375 | 675 | 900 | 150 |
| Lean beef (one steak) | 0 | 10.05 | 21.45 | 32.10 | 75.00 | 117.90 | 150 | 375 | 675 | 900 | 150 |
| Tomato (a tomato) | 0 | 13.40 | 28.60 | 42.80 | 100.00 | 157.20 | 200 | 500 | 900 | 1200 | 200 |
| Trout (one medium trout) | 0 | 10.05 | 21.45 | 32.10 | 75.00 | 117.90 | 150 | 375 | 675 | 900 | 150 |
| Grape (a dessert plate) | 0 | 10.72 | 22.88 | 34.24 | 80.00 | 125.76 | 160 | 400 | 720 | 960 | 160 |
| White / rosé wine (one glass. 100ml) | 0 | 6.70 | 14.30 | 21.40 | 50.00 | 78.60 | 100 | 250 | 450 | 600 | 100 |
| Red wine (one glass. 100ml) | 0 | 6.70 | 14.30 | 21.40 | 50.00 | 78.60 | 100 | 250 | 450 | 600 | 100 |
| Skimmed Milk yogurt (one unit) | 0 | 8.38 | 17.88 | 26.75 | 62.50 | 98.25 | 125 | 312.5 | 562.5 | 750 | 125 |
| Whole Milk yogurt (one unit) | 0 | 8.38 | 17.88 | 26.75 | 62.50 | 98.25 | 125 | 312.5 | 562.5 | 750 | 125 |
| Carrots (a normal dish) | 0 | 13.40 | 28.60 | 42.80 | 100.00 | 157.20 | 200 | 500 | 900 | 1200 | 200 |
| Peach juice (one glass. 200ml) | 0 | 13.40 | 28.60 | 42.80 | 100.00 | 157.20 | 200 | 500 | 900 | 1200 | 200 |
| Apple juice (one glass. 200ml) | 0 | 13.40 | 28.60 | 42.80 | 100.00 | 157.20 | 200 | 500 | 900 | 1200 | 200 |
| Orange juice (one glass. 200ml) | 0 | 13.40 | 28.60 | 42.80 | 100.00 | 157.20 | 200 | 500 | 900 | 1200 | 200 |
| Tomato juice (one glass. 200ml) | 0 | 13.40 | 28.60 | 42.80 | 100.00 | 157.20 | 200 | 500 | 900 | 1200 | 200 |

Source: Own elaboration with data from online price comparators of supermarkets located in the same geographical area as that of the population sample (carritus.com and soysuper.com).

Note: clarification on translating rations to daily amounts

| - 1-3 times / month (approximates like 2 times a month or 0.5 times a week) | ${Amount}_{i}=\frac{Portion}{2\cdot7}$ |
| --- | --- |
| - 1 time/week | ${Amount}_{i}=\frac{Portion}{7}$ |
| - 1-2 times / week (approximates about 1.5 times a week) | ${Amount}_{i}=\frac{1.5\cdot Portion}{7}$ |
| - 3-4 times /week (approximates about 3.5 times a week) | ${Amount}_{i}=\frac{3.5\cdot Portion}{7}$ |
| - 5-6 times /week (approximates about 5.5 times a week) | ${Amount}_{i}=\frac{5.5\cdot Portion}{7}$ |
| - 1 time / day | ${Amount}_{i}={Portion}_{i}$ |
| - 2-3 times /day (approximates about 2.5 times a day) | ${Amount}_{i}={2.5\cdot Portion}_{i}$ |
| - 4-5 times / day (approximates about 4.5 times a day) | ${Amount}_{i}={4.5\cdot Portion}_{i}$ |
| - 6 or more times / day | ${Amount}_{i}={6\cdot Portion}_{i}$ |

**Table A2. Descriptive statistics for the prices of all product categories**

|  | No. of references | Average price | Std. Dev. | Maximum price | Minimum price |
| --- | --- | --- | --- | --- | --- |
| Sunflower oil | 8 | 1.14 | 0.24 | 1.69 | 0.96 |
| Corn oil | 2 | 1.91 | 0.06 | 1.97 | 1.85 |
| Extra virgin olive oil | 7 | 2.54 | 0.31 | 2.99 | 2.19 |
| Olive oil | 11 | 5.62 | 1.08 | 7.99 | 4.50 |
| Seed oil | 4 | 2.10 | 0.12 | 2.29 | 1.98 |
| Swiss chard | 4 | 1.93 | 0.25 | 2.25 | 1.67 |
| Avocado | 4 | 6.47 | 2.00 | 8.50 | 4.00 |
| Clams | 5 | 3.97 | 0.58 | 4.67 | 2.95 |
| Shelled almonds | 5 | 17.64 | 1.98 | 20.25 | 15.45 |
| Anchovies in oil | 6 | 65.08 | 19.38 | 96.21 | 43.93 |
| White rice | 6 | 1.01 | 0.30 | 1.52 | 0.79 |
| Brown rice | 4 | 1.58 | 0.09 | 1.71 | 1.49 |
| Fresh Tuna | 2 | 12.95 | 2.00 | 14.95 | 10.95 |
| Tuna, mackerel, bonito in oil | 13 | 22.80 | 5.52 | 30.49 | 11.80 |
| Picked tuna, mackerel, or bonito | 3 | 11.37 | 0.41 | 11.83 | 10.83 |
| Shelled hazelnuts | 6 | 18.67 | 5.90 | 29.50 | 12.25 |
| Sugar | 6 | 0.76 | 0.03 | 0.79 | 0.69 |
| Bacon | 13 | 8.44 | 0.92 | 10.00 | 6.52 |
| Cockles | 16 | 29.30 | 11.29 | 54.95 | 11.60 |
| Eggplants | 3 | 1.85 | 0.10 | 1.99 | 1.78 |
| Hamburger | 1 | 4.00 | 0.00 | 4.00 | 4.00 |
| Broccoli | 3 | 2.86 | 0.05 | 2.90 | 2.78 |
| Mackerel or anchovy | 2 | 4.25 | 0.25 | 4.50 | 4.00 |
| Shelled peanuts | 6 | 3.13 | 0.09 | 3.28 | 2.98 |
| Cocoa | 3 | 5.89 | 0.50 | 6.24 | 5.18 |
| Coffee | 44 | 9.21 | 2.60 | 15.00 | 3.98 |
| Black coffee | 3 | 1.00 | 0.08 | 1.10 | 0.90 |
| Zucchini | 4 | 1.66 | 0.16 | 1.79 | 1.39 |
| Squid | 10 | 11.38 | 2.99 | 16.95 | 5.95 |
| Soup | 57 | 2.39 | 1.56 | 6.51 | 0.82 |
| Cannelloni | 31 | 5.80 | 1.75 | 9.97 | 3.32 |
| Fatty pork (ribs. chops) | 15 | 5.21 | 1.07 | 8.40 | 3.95 |
| Lean pork (tenderloin. sirloin) | 20 | 7.40 | 1.35 | 9.35 | 4.90 |
| Rabbit | 2 | 6.23 | 0.28 | 6.50 | 5.95 |
| Lamb meat | 6 | 10.07 | 1.86 | 12.90 | 6.90 |
| Beef (ribs. stew) | 9 | 8.11 | 2.84 | 14.90 | 4.95 |
| Lean beef (steak) | 4 | 13.57 | 4.89 | 21.00 | 8.96 |
| Beef | 6 | 10.93 | 3.51 | 15.90 | 6.30 |
| Sparkling wine | 79 | 7.57 | 4.43 | 21.67 | 2.60 |
| Onions | 8 | 1.20 | 0.33 | 1.85 | 0.83 |
| Cornflakes | 79 | 4.41 | 1.49 | 7.44 | 1.59 |
| Beer with alcohol | 50 | 2.20 | 0.65 | 3.64 | 1.09 |
| Non-alcoholic beer | 31 | 1.66 | 0.45 | 3.58 | 0.82 |
| Chocolate | 39 | 11.02 | 3.78 | 19.88 | 4.50 |
| Plums | 4 | 5.20 | 1.37 | 6.60 | 3.29 |
| Brussels sprout | 4 | 3.52 | 1.13 | 4.90 | 1.76 |
| Cabbage | 10 | 1.70 | 0.42 | 2.40 | 0.99 |
| Oatmeal | 4 | 2.35 | 0.74 | 3.40 | 1.54 |
| Croissant | 9 | 2.68 | 0.34 | 3.20 | 2.20 |
| Croquettes | 21 | 4.90 | 2.01 | 8.80 | 1.98 |
| Donut | 10 | 3.02 | 1.33 | 5.75 | 1.56 |
| Endives | 3 | 2.77 | 0.20 | 2.97 | 2.50 |
| Escarole | 3 | 1.35 | 0.14 | 1.45 | 1.15 |
| Fresh asparagus | 6 | 8.79 | 0.82 | 9.95 | 8.00 |
| Spinach | 12 | 3.34 | 1.48 | 6.22 | 1.13 |
| Fruits in syrup | 5 | 2.89 | 0.41 | 3.26 | 2.12 |
| Dates, raisins, dried figs | 7 | 6.24 | 1.88 | 10.60 | 4.30 |
| Cookies | 174 | 4.29 | 2.06 | 9.50 | 1.10 |
| Prawns | 17 | 14.32 | 2.69 | 17.98 | 7.95 |
| Chickpeas | 13 | 2.64 | 0.55 | 3.91 | 1.88 |
| Peas | 11 | 2.04 | 0.86 | 3.95 | 1.09 |
| Broad beans | 3 | 3.51 | 0.47 | 4.00 | 2.88 |
| Ice cream | 26 | 2.77 | 0.81 | 4.58 | 1.22 |
| Beef, pork, or lamb liver | 13 | 6.97 | 2.58 | 11.30 | 2.98 |
| Eggs | 20 | 0.17 | 0.07 | 0.37 | 0.08 |
| Cooked ham | 41 | 9.87 | 2.39 | 16.58 | 6.28 |
| Serrano ham | 35 | 16.16 | 5.22 | 31.30 | 7.24 |
| Green beans | 14 | 3.12 | 0.79 | 4.15 | 1.09 |
| Kiwis | 3 | 3.89 | 1.49 | 6.00 | 2.69 |
| Soy milk | 27 | 1.04 | 0.28 | 1.65 | 0.68 |
| Skim milk | 37 | 0.81 | 0.15 | 1.19 | 0.55 |
| Whole milk | 32 | 0.80 | 0.14 | 1.12 | 0.55 |
| Semi-skimmed milk | 37 | 0.83 | 0.16 | 1.19 | 0.56 |
| Lentils | 23 | 2.07 | 0.72 | 3.50 | 1.04 |
| Liquors | 74 | 10.79 | 5.09 | 22.64 | 3.27 |
| Lemons | 6 | 2.28 | 0.42 | 2.98 | 1.55 |
| Cupcake | 28 | 3.35 | 1.32 | 7.00 | 1.61 |
| Apple | 20 | 2.14 | 0.63 | 3.59 | 1.29 |
| Margarine | 21 | 3.76 | 1.61 | 6.60 | 1.60 |
| Mayonnaise | 35 | 3.85 | 0.99 | 6.26 | 1.89 |
| Mussels or clams | 5 | 4.43 | 0.94 | 5.63 | 3.00 |
| Peach | 6 | 2.89 | 0.36 | 3.50 | 2.29 |
| Melon | 6 | 2.18 | 0.41 | 2.58 | 1.59 |
| Blood sausage | 16 | 8.00 | 1.99 | 13.13 | 4.47 |
| Mortadella. chorizo | 75 | 7.22 | 3.30 | 14.92 | 2.02 |
| Muesli | 14 | 3.22 | 0.71 | 4.50 | 1.59 |
| Orange | 12 | 1.32 | 0.44 | 2.00 | 0.80 |
| Whipped cream, heavy cream | 12 | 3.57 | 1.12 | 6.00 | 2.10 |
| Custard, flan, pudding | 23 | 2.38 | 0.96 | 5.28 | 1.32 |
| Bread | 20 | 2.03 | 0.54 | 3.10 | 1.43 |
| Sliced white bread | 26 | 2.75 | 1.07 | 4.54 | 1.16 |
| Sliced whole wheat bread | 25 | 3.64 | 1.70 | 7.50 | 1.16 |
| Pasta | 47 | 1.77 | 0.79 | 4.10 | 0.75 |
| Whole wheat pasta | 14 | 2.91 | 1.05 | 5.38 | 1.78 |
| Cake | 35 | 3.85 | 1.64 | 7.77 | 1.33 |
| Potatoes | 14 | 1.22 | 0.24 | 1.60 | 0.90 |
| Potato chips (a normal bag) | 54 | 6.85 | 2.03 | 10.80 | 3.06 |
| Pate / foie gras | 33 | 6.72 | 2.61 | 13.00 | 2.13 |
| Chicken or turkey breast without skin | 14 | 6.69 | 1.00 | 8.50 | 4.60 |
| Cucumber | 6 | 1.98 | 0.42 | 2.60 | 1.27 |
| Pear | 7 | 2.07 | 0.70 | 3.69 | 1.49 |
| Peppers | 10 | 2.92 | 0.61 | 3.95 | 1.79 |
| Pistachios | 7 | 12.59 | 1.30 | 15.75 | 11.88 |
| Banana | 6 | 2.21 | 0.65 | 3.29 | 1.29 |
| Blue cheese | 14 | 14.13 | 5.09 | 29.23 | 9.27 |
| Brie, Camembert | 12 | 11.97 | 3.56 | 15.00 | 5.21 |
| Gruyere / Emmental / gouda | 13 | 6.78 | 1.30 | 9.40 | 5.65 |
| Cottage cheese. or other soft and fresh cheese | 19 | 5.91 | 1.75 | 8.76 | 3.10 |
| Hard cheese | 13 | 14.30 | 2.15 | 17.70 | 10.42 |
| Carbonated drinks- Soft drink: Coke, Fanta and similar | 88 | 0.70 | 0.32 | 1.78 | 0.25 |
| Beef, pork or lamb kidneys | 2 | 9.90 | 6.00 | 15.90 | 3.90 |
| Salt | 13 | 0.28 | 0.06 | 0.40 | 0.22 |
| Salami, salchichón | 30 | 10.48 | 2.41 | 13.88 | 6.00 |
| Salmon | 10 | 17.04 | 5.91 | 27.45 | 9.60 |
| Watermelon | 5 | 1.82 | 0.72 | 3.09 | 1.06 |
| Sardines | 1 | 7.50 | 0.00 | 7.50 | 7.50 |
| Sardines in oil | 22 | 14.00 | 5.23 | 23.00 | 4.58 |
| Pickled sardines | 4 | 9.21 | 3.97 | 12.26 | 2.56 |
| Mushrooms | 13 | 5.02 | 1.20 | 7.10 | 3.41 |
| Fried tomato, ketchup | 45 | 2.09 | 0.78 | 4.22 | 0.83 |
| Tomato | 15 | 2.17 | 0.83 | 3.64 | 1.30 |
| Trout | 2 | 9.78 | 3.28 | 13.05 | 6.50 |
| Grapes | 4 | 3.91 | 1.42 | 5.40 | 1.79 |
| White / rosé wine | 77 | 5.05 | 2.65 | 11.93 | 1.20 |
| Red wine | 130 | 5.70 | 3.22 | 14.93 | 1.08 |
| Skimmed Milk yogurt | 59 | 2.50 | 1.32 | 7.56 | 1.03 |
| Whole Milk yogurt | 149 | 2.35 | 1.16 | 6.77 | 0.96 |
| Carrots | 5 | 1.49 | 0.65 | 2.12 | 0.69 |
| Apple juice | 19 | 1.04 | 0.38 | 2.20 | 0.58 |
| Peach juice | 13 | 1.05 | 0.46 | 2.15 | 0.55 |
| Orange juice | 36 | 1.50 | 0.60 | 2.94 | 0.69 |
| Tomato juice | 7 | 1.39 | 0.68 | 2.35 | 0.65 |

Note: Prices are as € / litre and € / kg

**Table A3. Comparison of evolution of food and beverages prices and average disposable income in the period 2008-2019.**

|  | Spain | | | Badajoz | | |
| --- | --- | --- | --- | --- | --- | --- |
|  | 2008 | 2019 | AAAR | 2008 | 2019 | TMAA (%) |
| IPC |  |  |  |  |  |  |
| Food | 92.991 | 103.923 | 1.016 | 93.127 | 103.00 | 0.920 |
| Non-alcoholic beverages | 90.916 | 104.396 | 1.265 | 92.109 | 104.19 | 1.127 |
| Alcoholic drinks | 91.438 | 105.979 | 1.351 | 94.126 | 106.658 | 1.143 |
| Average disposable income (€ / year) | 20.658 | 22.634 | 0.834 | 15.005 | 17.439 | 1.376 |

AAAR: accumulated average annual rate.
Source: Own elaboration with data from the Spanish Statistics National Institute and the Statistical System of Extremadura Statistical System of Extremadura - Citizen Portal (gobex.es)

**Table A4. Comparison of the weight that food and beverages represent in the shopping basket.**

|  | Spain | | Badajoz | |  |
| --- | --- | --- | --- | --- | --- |
|  | Food and non-alcoholic beverages | Alcoholic drinks | Food and non-alcoholic beverages | Alcoholic drinks | Difference Badajoz - Spain |
| 2008 | 188.834 | 28.979 | 228.034 | 36.332 | 39.20 |
| 2009 | 202.796 | 26.678 | 245.063 | 33.639 | 42.27 |
| Weight CPI 2019 - Weight CPI 2008 | 13.962 | -2.301 | 17.029 | -2.693 |  |

Source: Own elaboration with data from the Spanish Statistics National Institute
